# Supplementary material for: Voltage-Gated Sodium Channel NaV1.7 Inhibitors with Potent Anticancer Activities in Medullary Thyroid Cancer Cells
Source: Cancers (Basel). 2023 May 17;15(10):2806. doi: 10.3390/cancers15102806 (PMC10216335; doi:10.3390/cancers15102806)
Supplement: Supplementary file 1 [file cancers-15-02806-s001.zip › cancers-2311277-supplementary.pdf]

Supplemental Information

Voltage-gated Sodium Channel NaV1.7 Inhibitors with Potent Anticancer Activities in Medullary Thyroid Cancer Cells

Piyasuda Pukkanasut[1], Jason Whitt[2], Rachael Guenter[2], Shannon E Lynch[3,5], Carlos Gallegos[4,5], Margarita Jacaranda Rosendo-Pineda[8], Juan Carlos Gomora[8], Herbert Chen[2,6], Diana Lin[6], Anna Sorace[4,5,7], Renata Jaskula-Sztul[2,7]* and Sadanandan E. Velu[1,7]*

[1]Department of Chemistry, [2]Department of Surgery, [3]Graduate Biomedical Sciences, [4]Department of Biomedical Engineering, [5]Department of Radiology, [6]Department of Pathology, [7]O'Neal Comprehensive Cancer Center, The University of Alabama at Birmingham, Birmingham, AL 35226, [7]Departamento de Neuropatología Molecular, Instituto de Fisiología Celular, Universidad Nacional Autónoma de México, 04510, Mexico City, Mexico.

* Corresponding authors:
Sadanandan E. Velu, PhD, Phone: (205) 975-2478, Email: svelu@uab.edu
Renata Jaskula-Sztul, PhD, Phone: (205) 975-3507, Email: rjsztul@uabmc.edu

Table of contents.

Figure S1. TMA quantification S3

Figure S2. Correlations plot between %Nav1.7 expression and patient disease status S4

Table S1. Point-biserial correlation between %Nav1.7 expression and patient disease status (normal thyroid, primary, and metastases) S4

Figure S3. Stronger block of Nav1.7 channel outward currents by SV188 S5

Copies of ^{1}H NMR and ^{13}C NMR spectra.

4,4 Diphenylbutyricacid (**2**) S6

3-(Piperidin-1-yl)propan-1-amine (**5**) S7

4,4-Diphenyl-N-[3-(piperidin-1-yl)propyl]butanamide (**6**) S8

(4,4-Diphenylbutyl)[3-(piperidin-1-yl)propyl]amine hydrochloride (**SV188**) S9

4,4-Diphenyl-N-(3-phenylpropyl)butanamide (**8**) S10

4,4-Diphenylbutyl(3-phenylpropyl)amine hydrochloride (**WJB-133**) S11

4-(4-Fluorophenyl)butyl[3-(piperidin-1-yl)propyl]amine hydrochloride (compound **4**) S12

HPLC traces

Figure S4. HPLC trace of SV188 hydrochloride S13

Figure S5. HPLC trace of WJB133 hydrochloride S14

Figure S6. HPLC trace of Compound 4 hydrochloride S15

Original images supporting all blot and gel results.

Figure S7. Original images and signal densities of the blots in Figure 3A of the manuscript S16

Figure S8. Original images and signal densities of the blots in Figure 3B of the manuscript. S17

Figure S9. Original images and signal densities of the blots in Figure 3C of the manuscript. S18-S19

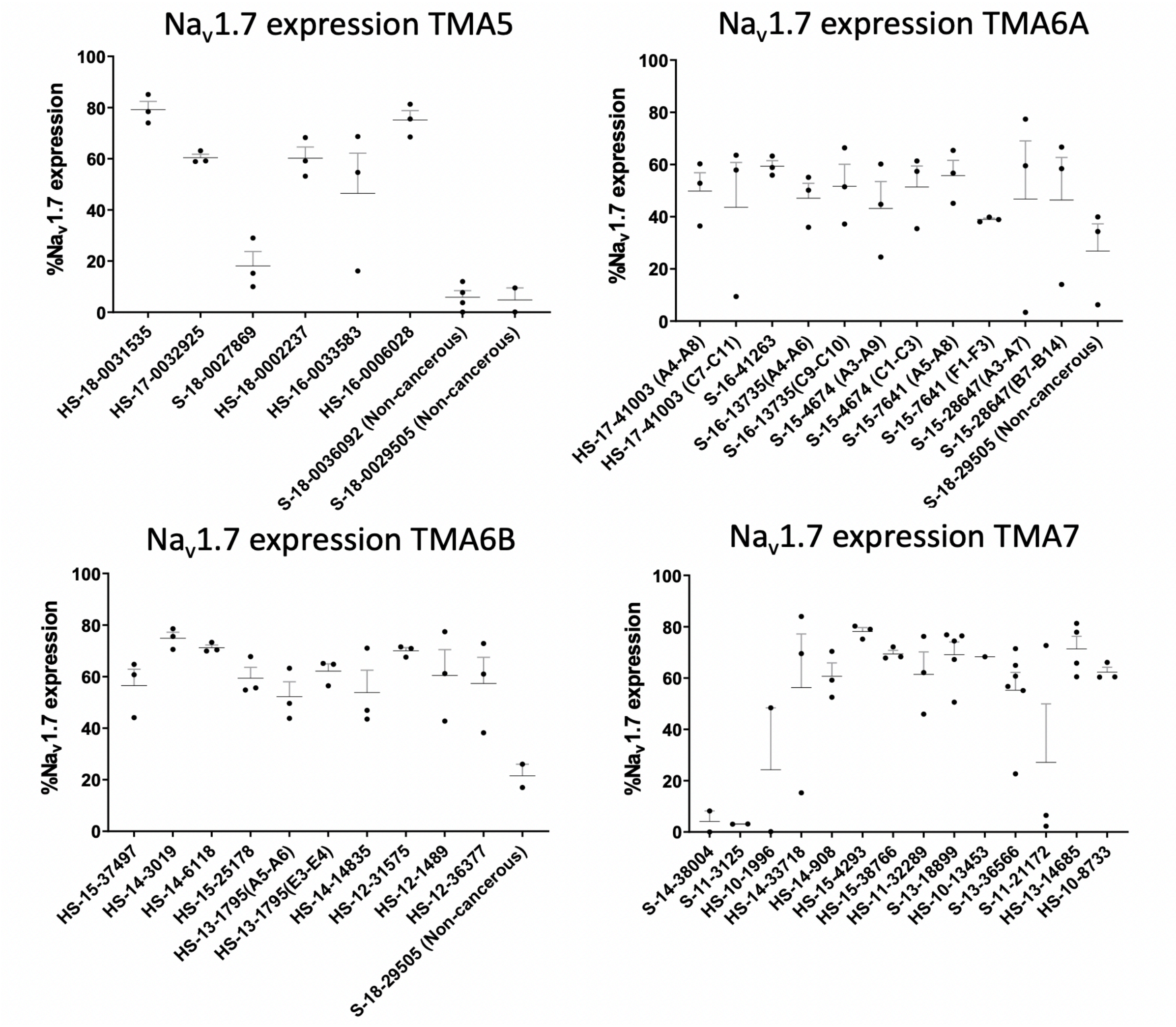

Supplementary Figure S1: TMA quantification from 45 individuals showing Nav1.7 expression in each tissue core with mean expression and standard error of the mean (SEM) of each subject on TMA slides, some individuals provided both primary and metastatic tissue cores.

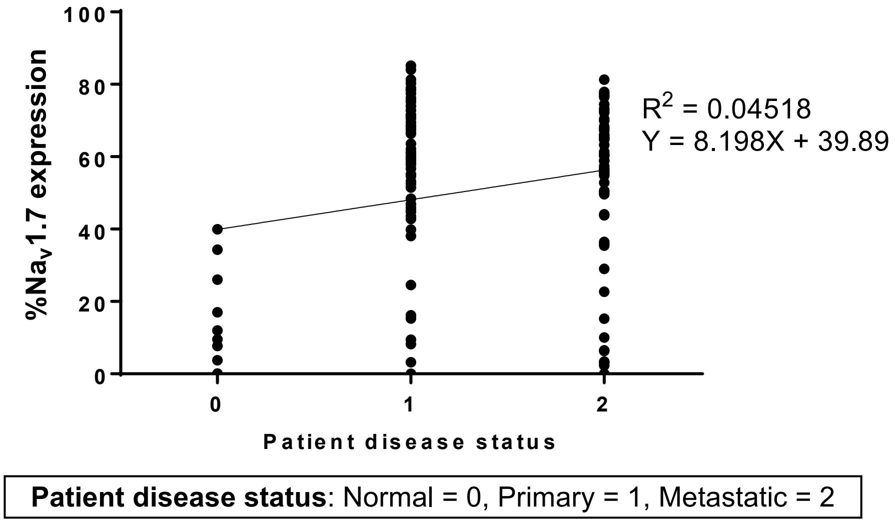

Supplementary Figure S2: Correlations plot between %Nav1.7 expression and patient disease status (0 = normal thyroid, 1 = primary and 2 = metastases). There was a positive correlation between %Nav1.7 expression and patient disease status (normal thyroid, primary and metastatic) with linear regression equation; Y = 8.198X + 39.89 and R^2 = 0.04518

Supplementary Table S1: Point-biserial correlation between %Nav1.7 expression and patient disease status (normal thyroid, primary, and metastases).

Correlations

		Disease status	%Nav1.7
%Nav1.7		.213*	--
	Sig. (2-tailed)	.014	
		133	133

*. Correlation is significant at the 0.05 level (2-tailed).

Confidence Intervals

	Pearson Correlation	Sig. (2-tailed)	95% Confidence Intervals (2-tailed)[a]	
			Lower	Upper
Disease status - %Nav1.7	.213	.014	.044	.369

a. Estimation is based on Fisher's r-to-z transformation.

Point-biserial correlation was run to determine the relationship between percent Nav1.7 expression and patient disease status (normal thyroid, primary, and metastases). There was a positive correlation between percent Nav1.7 expression and patient disease status which was statistically significant*, p = 0.014, rpb = 0.213, N (No. of cores) = 133 with 95% confidential interval = 95% CI [0.044, 0.369].

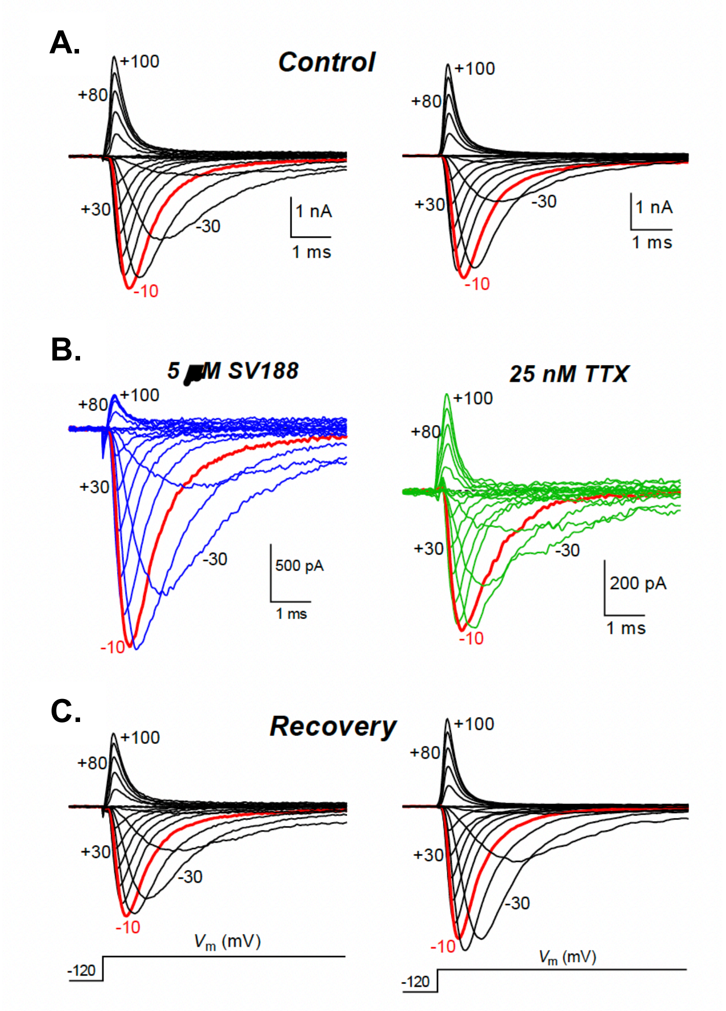

Figure S3: Stronger block of Nav1.7 channel outward currents by SV188. Representative families of Nav1.7 sodium currents obtained before (Control), during and after (Recovery) exposure to 5 μM of **SV188** (left panels) or 25 nM **TTX** (right panels). Currents were recorded in response to 16-ms depolarizing pulses from −60 to +80 mV in 10-mV steps applied every 5 s from a HP of −120 mV. Note that in the presence of **SV188** outward currents are strongly blocked, in comparison with those recorded in the presence of **TTX**.

Copies of ^{1}H NMR and ^{13}C NMR spectra

4,4-Diphenylbutyricacid (2)

^{1}H NMR ($CDCl_3$, 400 MHz)

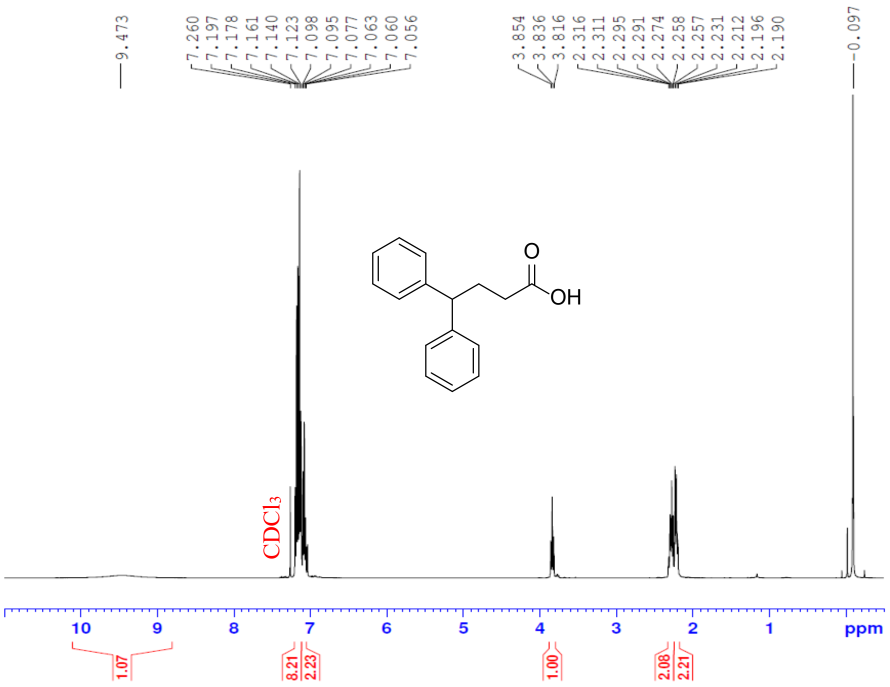

4,4-Diphenylbutyricacid (2)

^{13}C NMR ($CDCl_3$, 400 MHz)

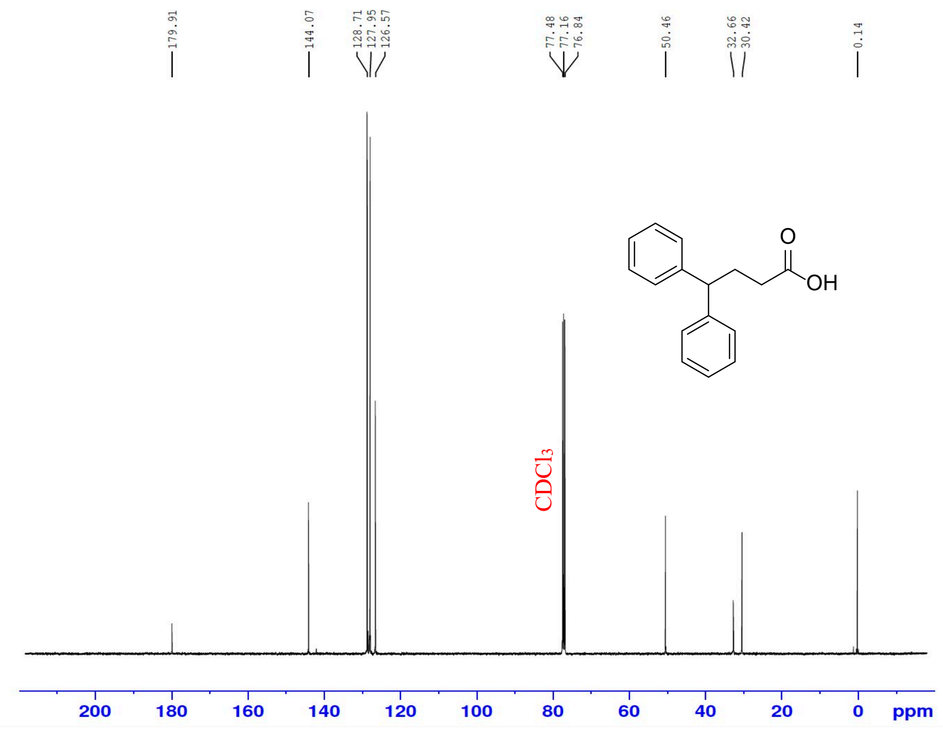

3-(Piperidin-1-yl)propan-1-amine (5)

^{1}H-NMR ($CDCl_3$, 400 MHz)

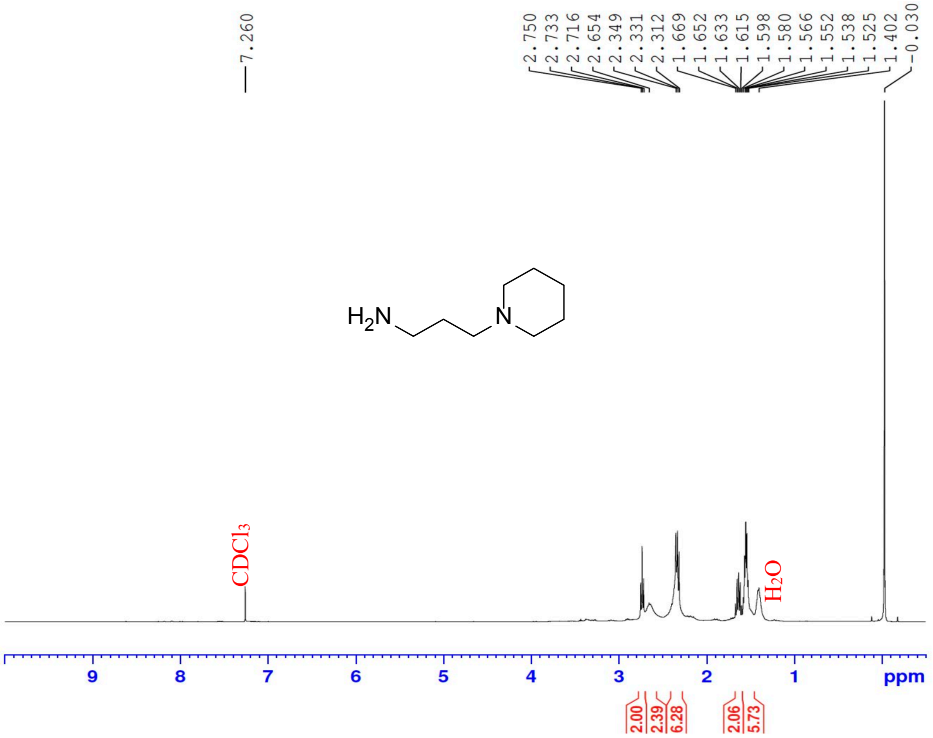

3-(Piperidin-1-yl)propan-1-amine (5)

^{13}C-NMR ($CDCl_3$, 400 MHz)

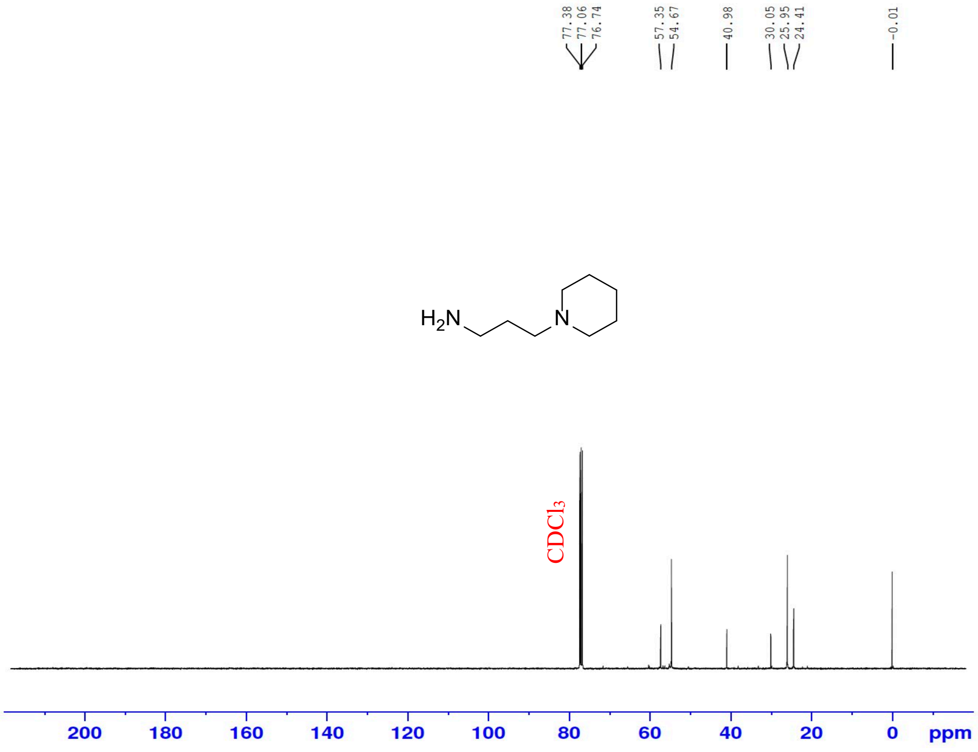

4,4-Diphenyl-N-[3-(piperidin-1-yl)propyl]butanamide (6)

^{1}H-NMR ($CDCl_3$, 400 MHz)

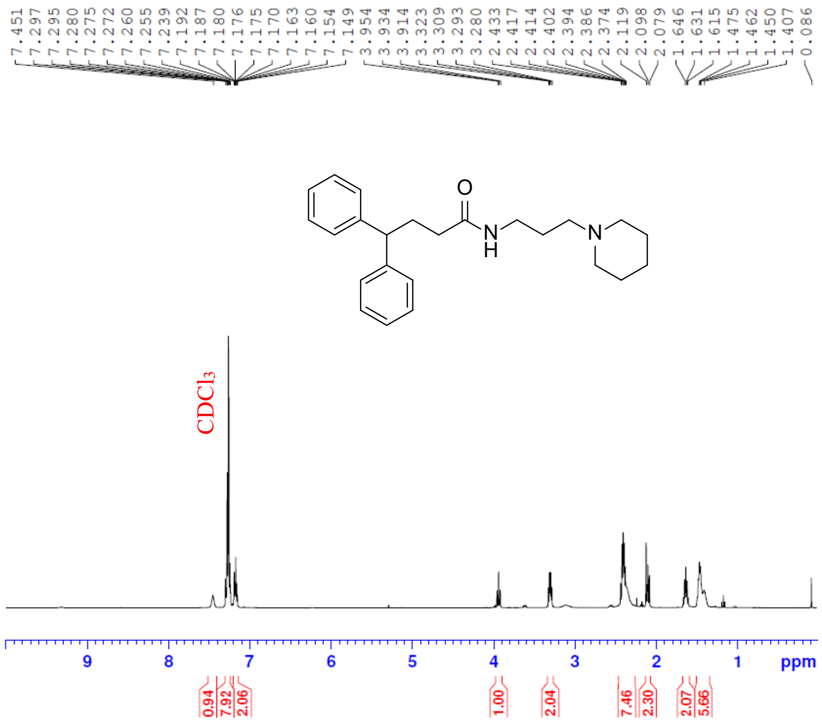

4,4-Diphenyl-N-[3-(piperidin-1-yl)propyl]butanamide (6)

^{13}C-NMR ($CDCl_3$, 400 MHz)

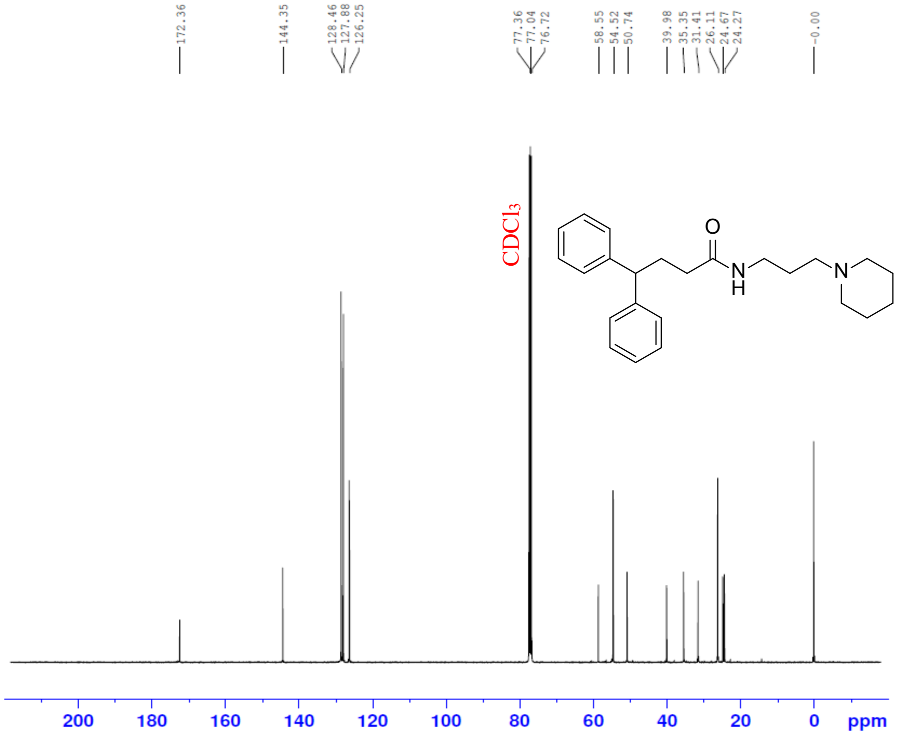

4,4-Diphenylbutyl[3-(piperidin-1-yl)propyl]amine hydrochloride (SV188)

^{1}H-NMR (DMSO-d$_6$, 400 MHz)

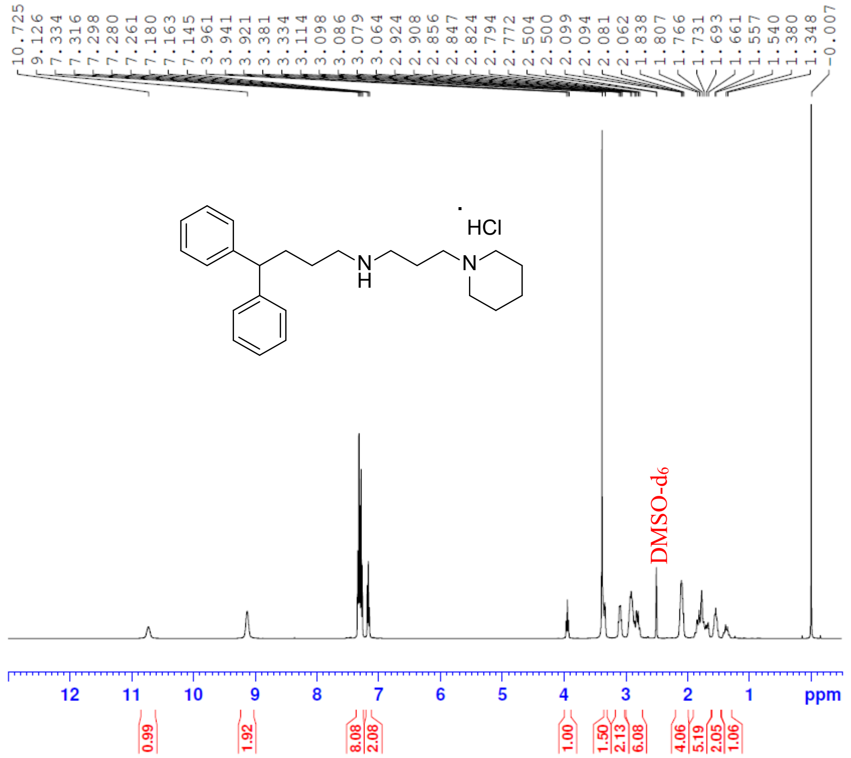

(4,4-Diphenylbutyl)[3-(piperidin-1-yl)propyl]amine hydrochloride (SV188)

^{13}C-NMR (DMSO-d$_6$, 400 MHz)

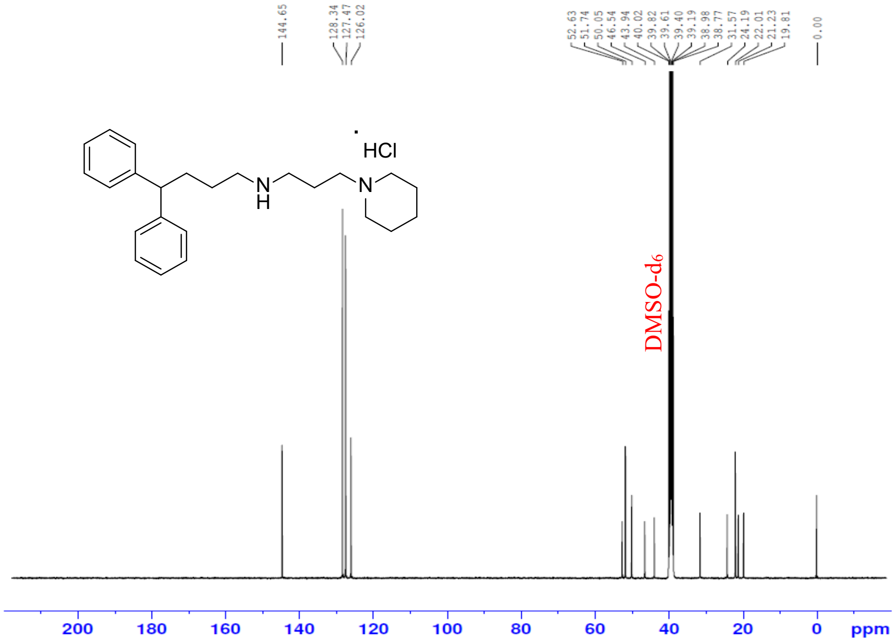

4,4-Diphenyl-N-(3-phenylpropyl)butanamide (8)

^1H-NMR ($CDCl_3$, 400 MHz)

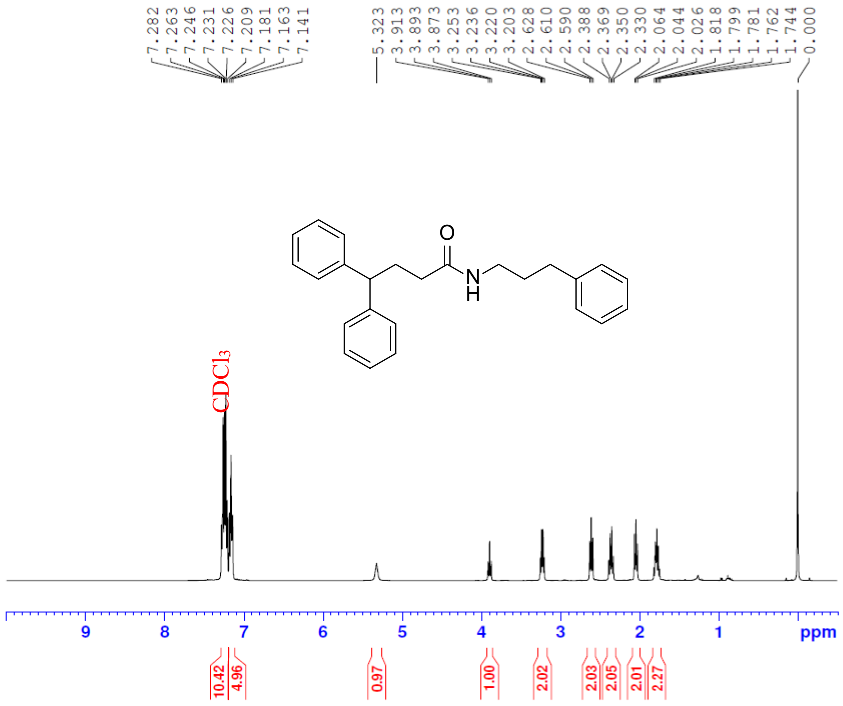

4,4-Diphenyl-N-(3-phenylpropyl)butanamide (8)

^{13}C-NMR ($CDCl_3$, 400 MHz)

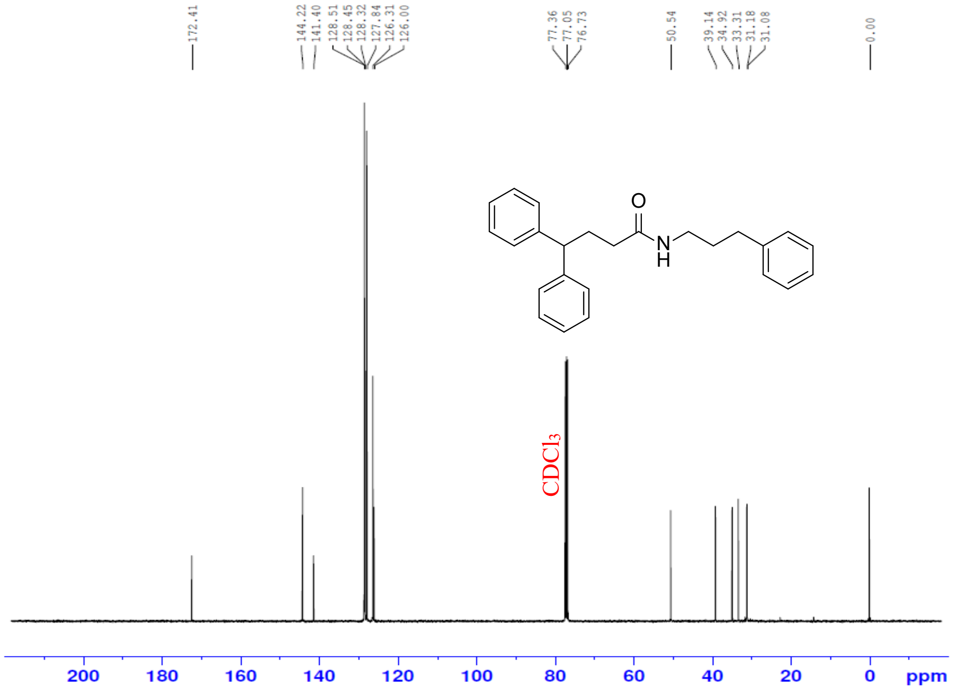

4,4-Diphenylbutyl(3-phenylpropyl)amine hydrochloride (WJB-133)

^{1}H-NMR ($CDCl_3$, 400 MHz)

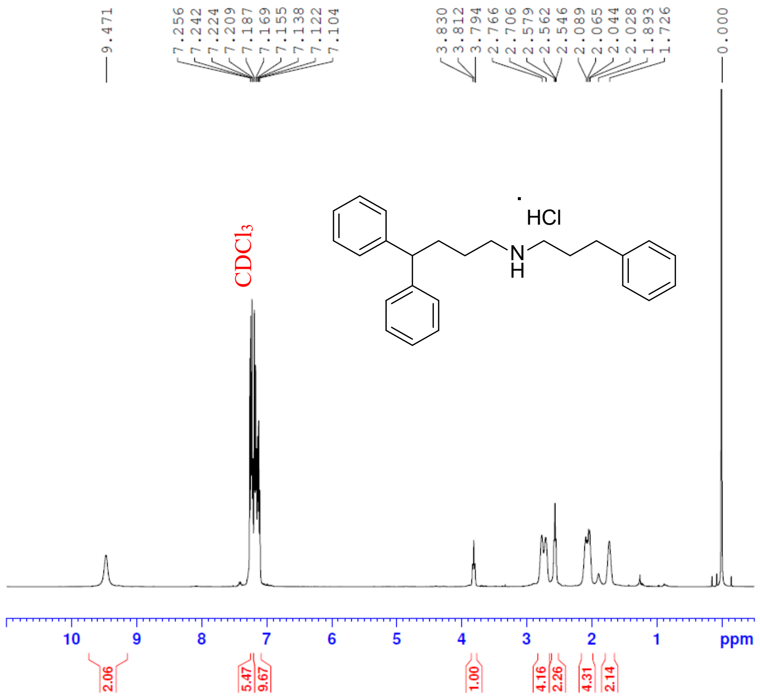

4,4-Diphenylbutyl(3-phenylpropyl)amine hydrochloride (WJB-133)

^{13}C-NMR ($CDCl_3$, 400 MHz)

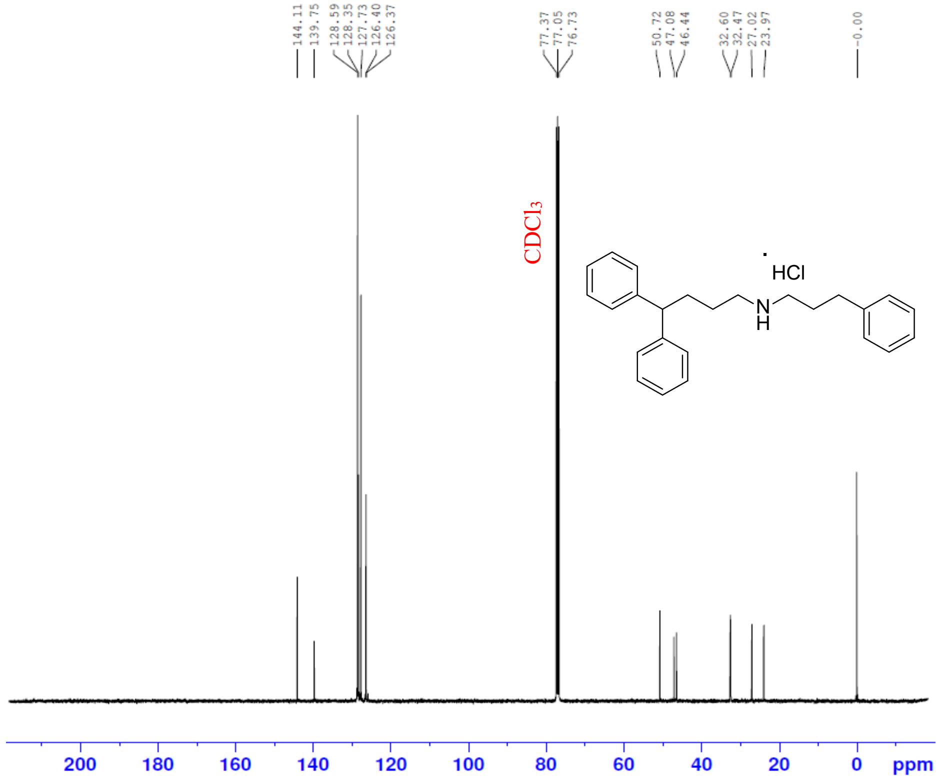

4-(4-Fluorophenyl)butyl][3-(piperidin-1-yl)propyl amine hydrochloride (compound 4)

^{1}H-NMR (DMSO-d$_6$, 400 MHz)

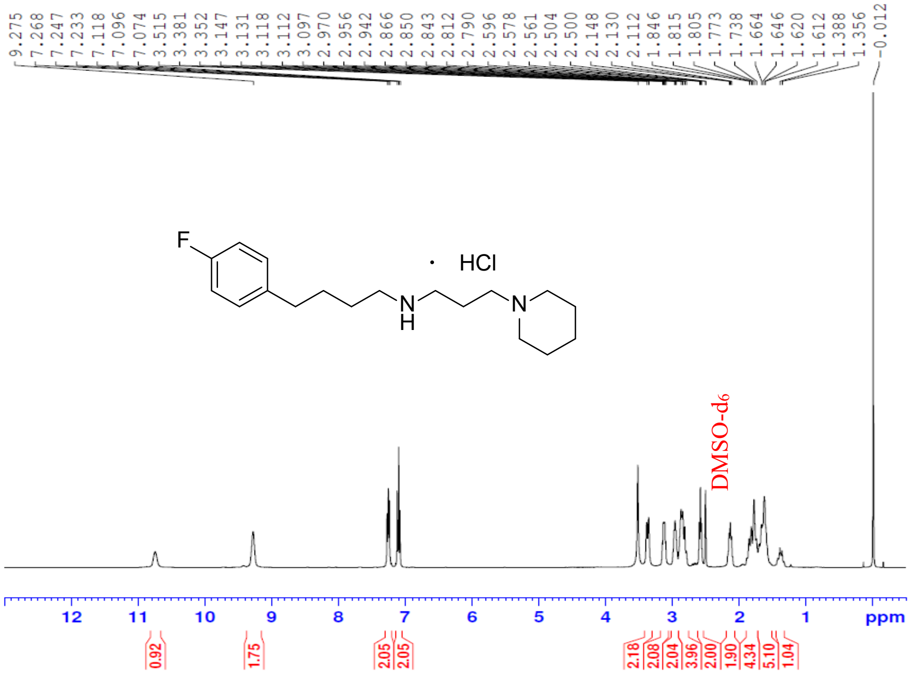

4-(4-Fluorophenyl)butyl][3-(piperidin-1-yl)propyl amine hydrochloride (compound 4)

^{13}C-NMR (DMSO-d$_6$, 400 MHz)

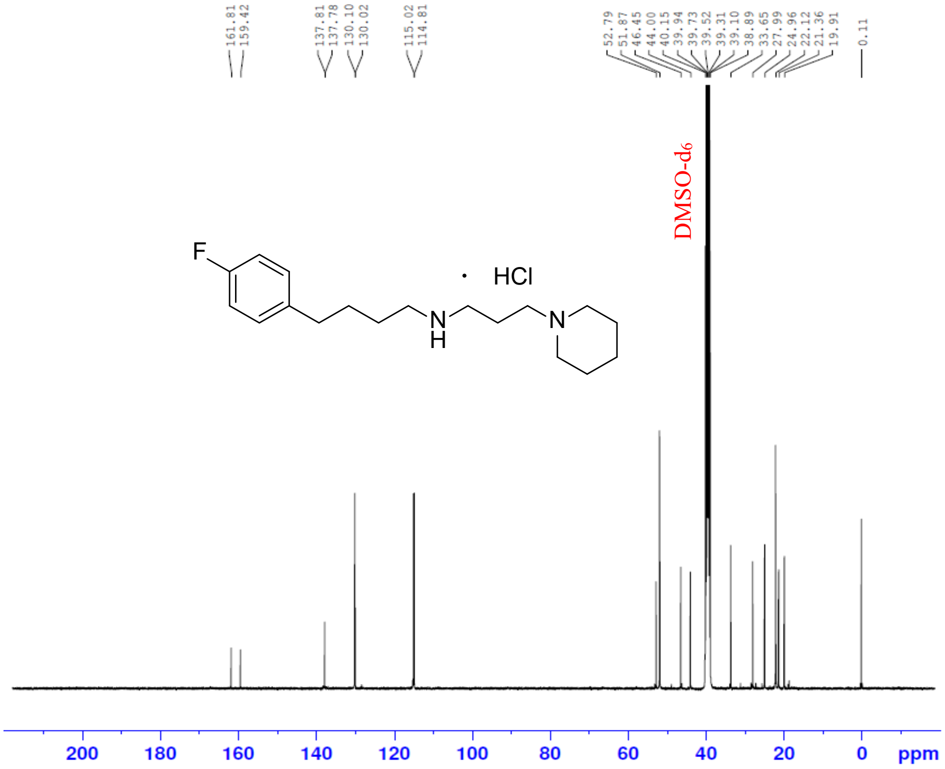

HPLC traces

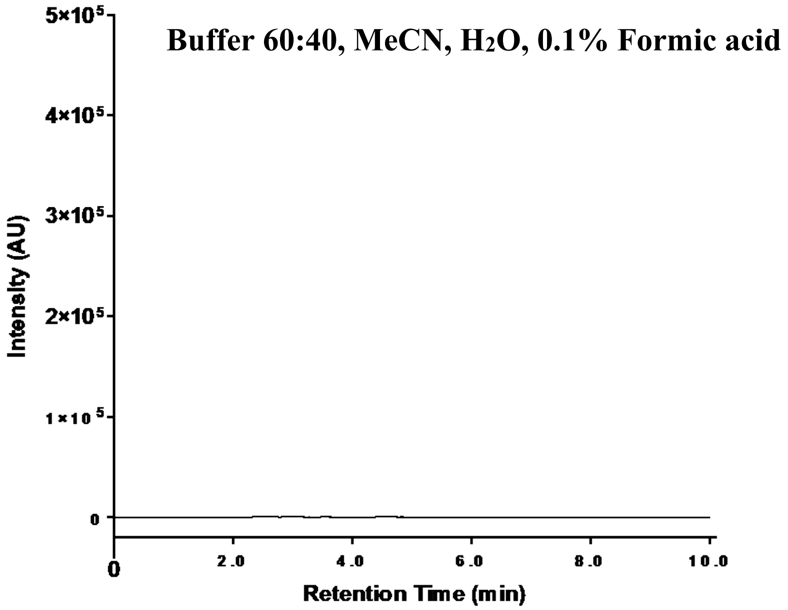

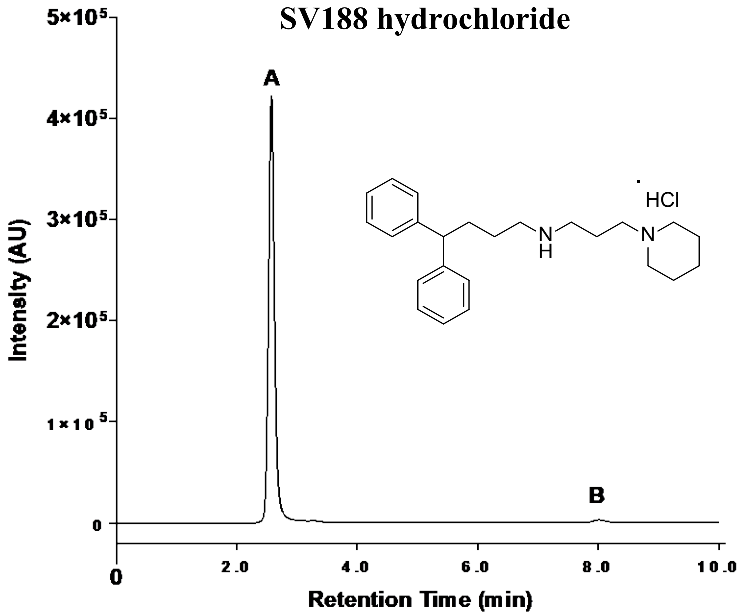

Compound	Peak	Retention Time (min)	Height (AU)	Area (AU*sec)	Area%
SV188	1	2.569	421710	2929025	97.897
	2	8.008	2859	62921	2.103

Figure S4: HPLC analysis of (4,4-diphenylbutyl)[3-(piperidin-1-yl)propyl]amine hydrochloride (SV188) using Kinetex 5 µm C18 100 Å, LC Column 150 x 4.6 mm, [SV188] = 3 mM, 20 µL injection, solvent: mobile phase buffer, Conditions: 60% MeCN/40% H_2O/0.1% Formic acid (isocratic), HPLC method 0-10 min,

Signals were analyzed using a 254 nm UV detector. A chromatogram of Mobile Phase Buffer (20 µL) was obtained for comparison.

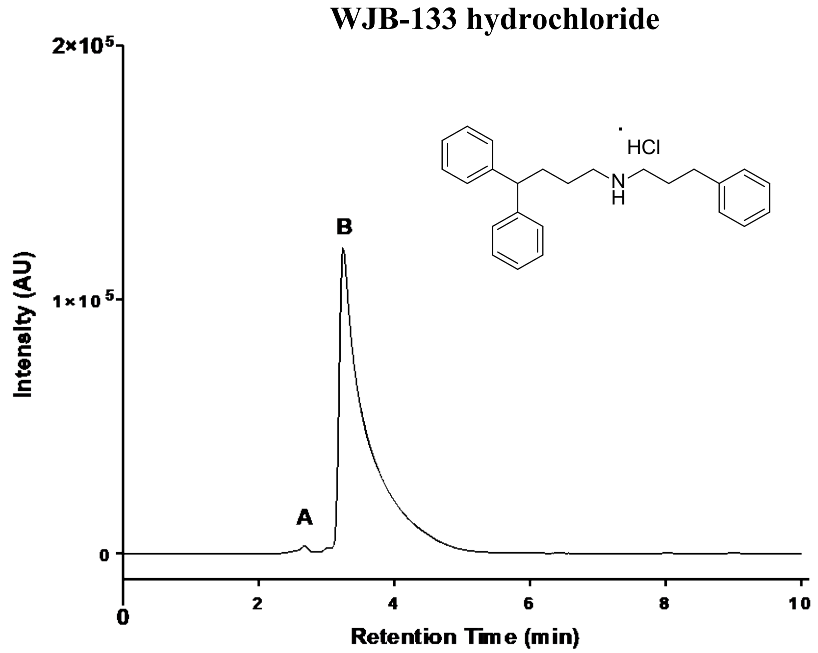

Compound	Peak	Retention Time (min)	Height (AU)	Area (AU*sec)	Area%
WJB-133	1	2.669	2803	28164	0.8345
	2	3.241	119527	3346628	99.1655

Figure S5: HPLC analysis of (4,4-Diphenylbutyl)(3-phenylpropyl)amine hydrochloride (WJB-133) using Kinetex 5 µm C18 100 Å, LC Column 150 x 4.6 mm, [WJB-133] = 3 mM, 20 µL injection, solvent: mobile phase buffer, Conditions: 60% MeCN/40% H_2O/0.1% Formic acid (isocratic), HPLC method 0-10 min, Signals were analyzed using a 254 nm UV detector. A chromatogram of Mobile Phase Buffer (20 µL) was obtained for comparison.

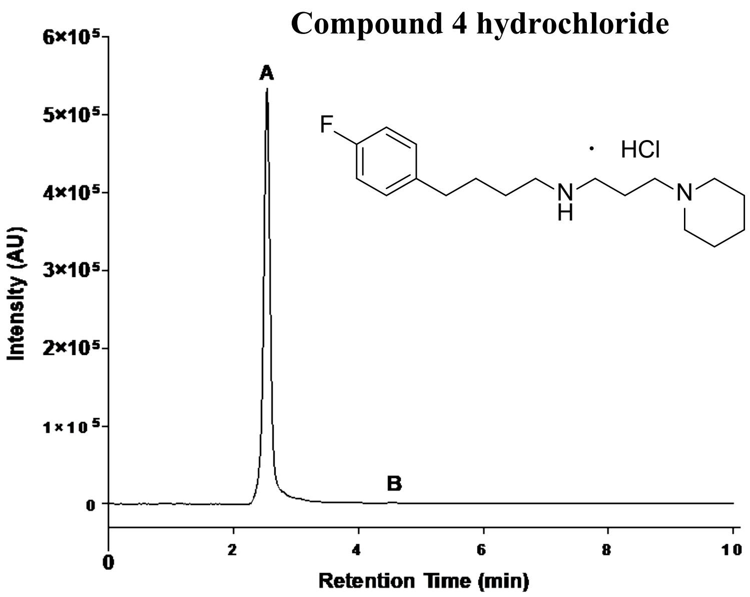

Compound	Peak	Retention Time (min)	Height (AU)	Area (AU*sec)	Area%
Compound 4	1	2.531	533682	4203875	99.7724
	2	4.549	838	9591	0.2276

Figure S6: HPLC analysis of 4-(4-fluorophenyl)butyl][3-(piperidin-1-yl)propyl amine hydrochloride (compound 4) using Kinetex 5 μm C18 100 Å, LC Column 150 x 4.6 mm, [compound 4] = 3 mM, 20 μL injection, solvent: mobile phase buffer, Conditions: 60% MeCN/40% H_2O/0.1% Formic acid (isocratic), HPLC method 0-10 min, Signals were analyzed using a 254 nm UV detector. A chromatogram of Mobile Phase Buffer (20 μL) was obtained for comparison.

<u>**Original images supporting all blot and gel results.**</u>

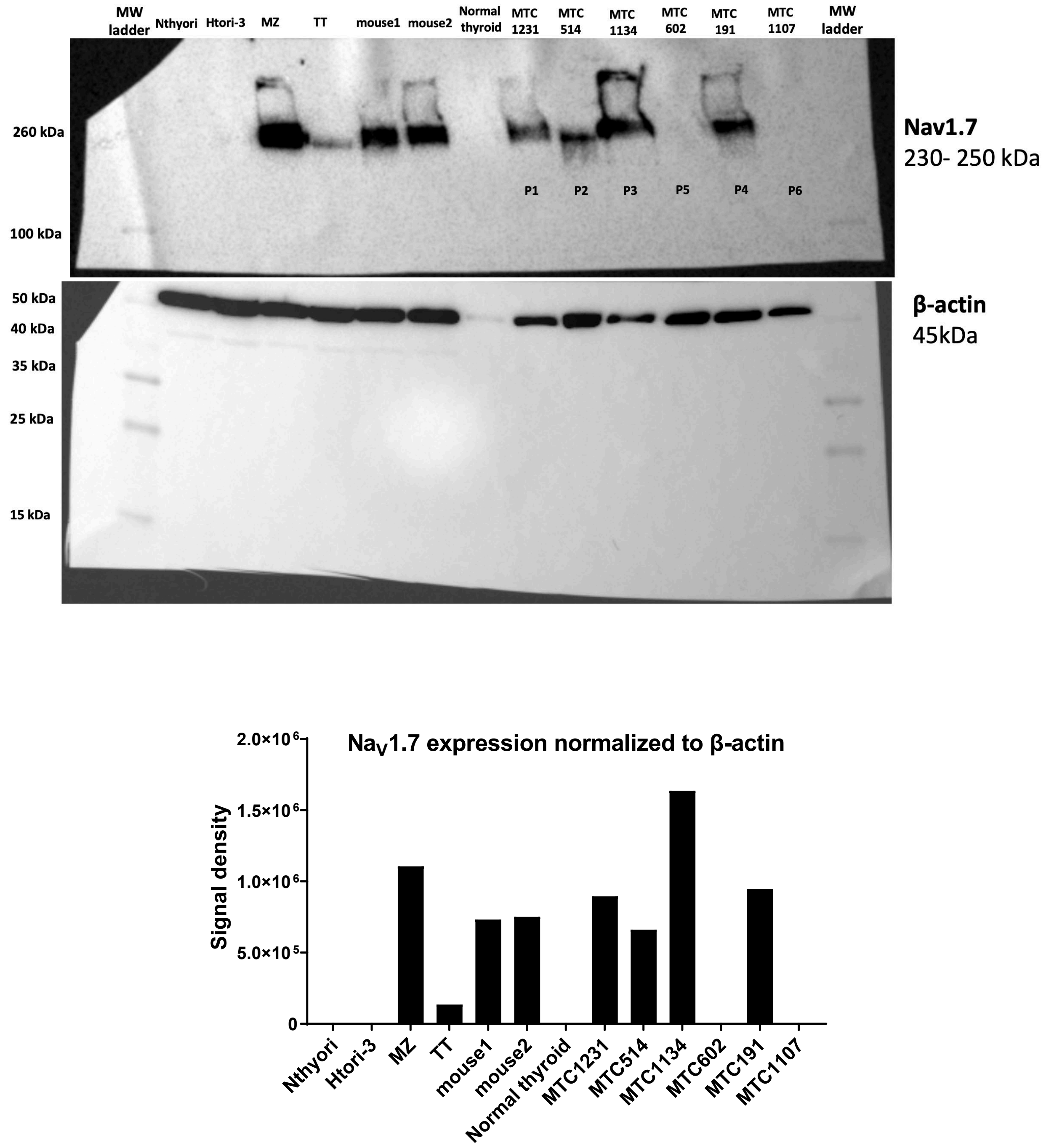

Figure S7: Original images and signal densities of the blots in Figure 3A of the manuscript. Nav1.7 was detected in human MTC cell lines, MZ-CRC-1 and TT, transgenic MTC mouse cell lines compared to normal human thyroid cell lines, Nthyori and Htori-3, which showed no detectable expression of Nav1.7.

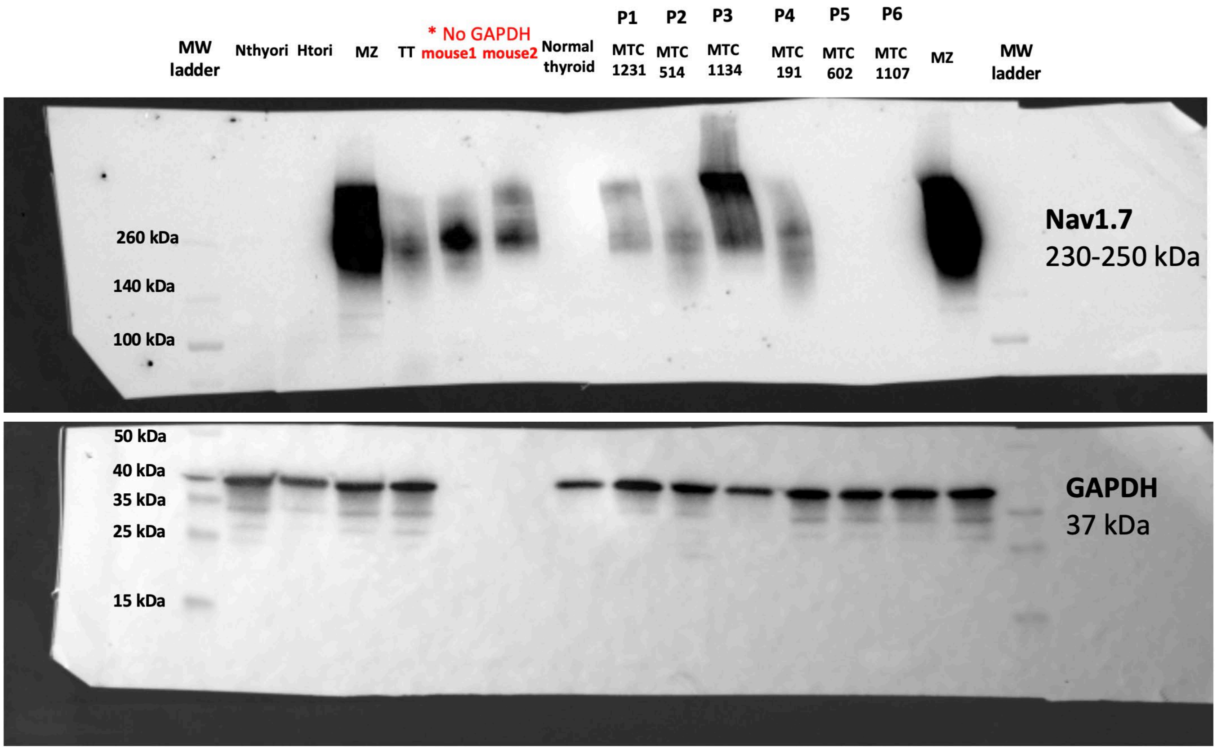

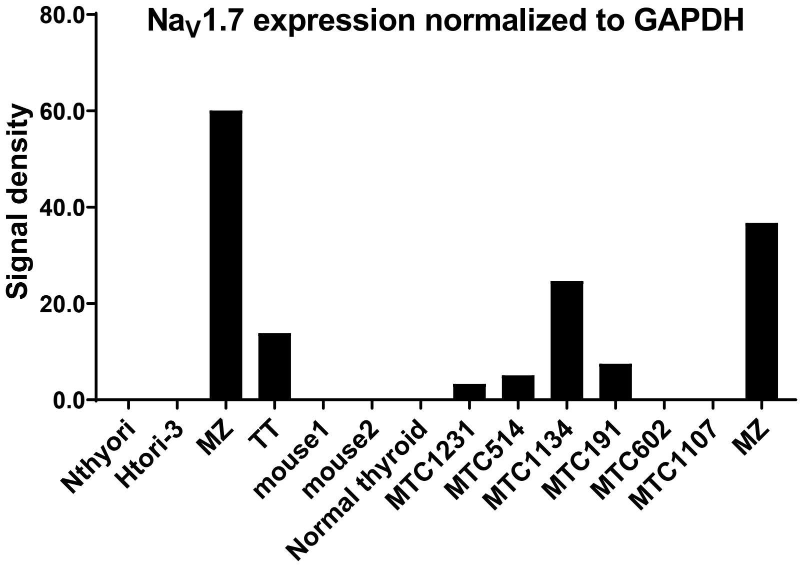

Figure S8: Original images and signal densities of the blots in of Figure 3B of the manuscript. Four out of six MTC patient tissues showed the expression of Nav1.7 while it was not detected in normal thyroid tissue.

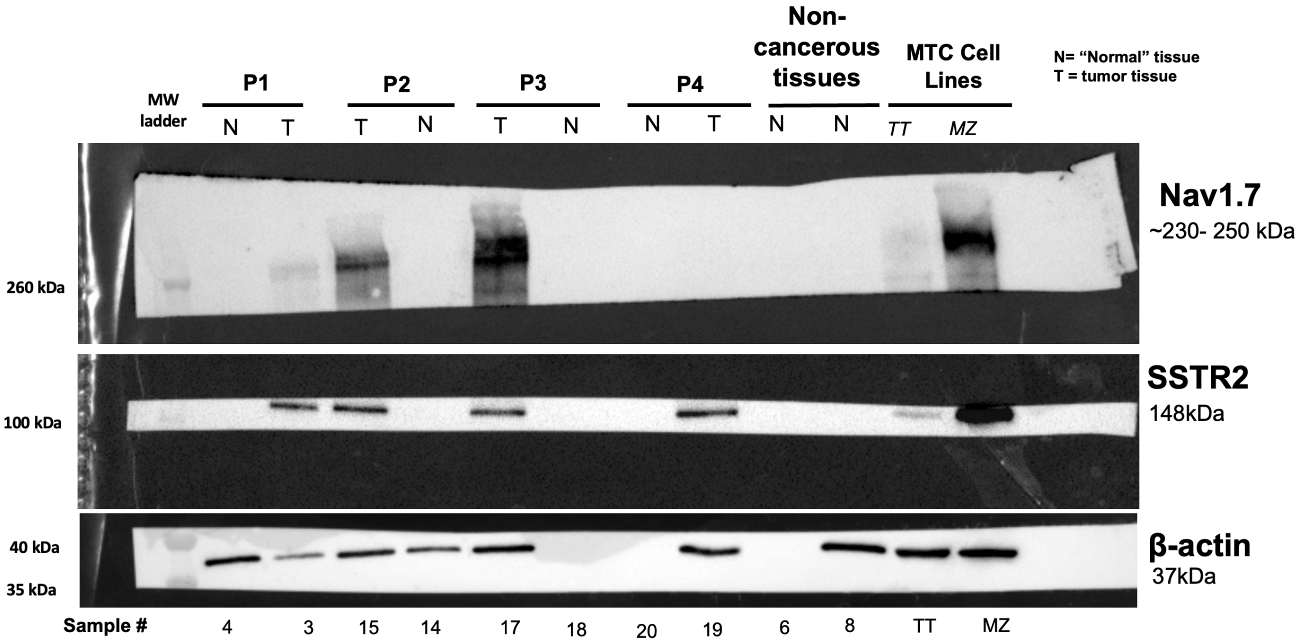
MW ladder
P1
P2
P3
P4
Non-cancerous tissues
MTC Cell Lines
N= "Normal" tissue
T = tumor tissue
N T T N T N N T N N TT MZ
Nav1.7
~230- 250 kDa
260 kDa
SSTR2
148kDa
100 kDa
β-actin
37kDa
40 kDa
35 kDa
Sample #
4 3 15 14 17 18 20 19 6 8 TT MZ

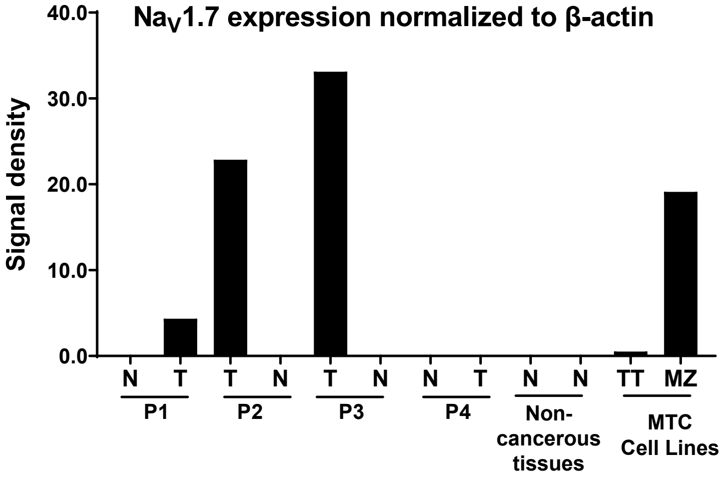
Na$_V$1.7 expression normalized to β-actin
Signal density
40.0
30.0
20.0
10.0
0.0
N T T N T N N T N N TT MZ
P1
P2
P3
P4
Non-cancerous tissues
MTC Cell Lines

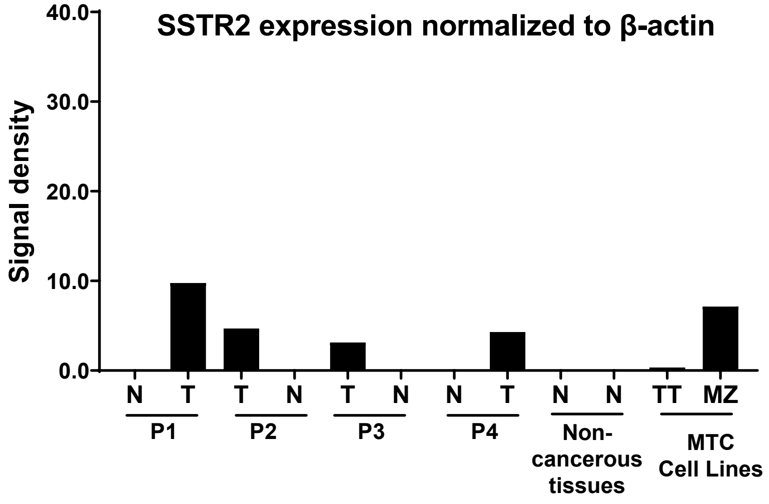
SSTR2 expression normalized to β-actin
Signal density
40.0
30.0
20.0
10.0
0.0
N T T N T N N T N N TT MZ
P1
P2
P3
P4
Non-cancerous tissues
MTC Cell Lines

Figure S9: Original images and signal densities of the blots in Figure 3C of the manuscript. A comparison of SSTR2 expression (neuroendocrine cancer biomarker) with Nav1.7 expression in normal tissues and MTC patient tissues. SSTR2 expression exhibited matched trends with Nav1.7 expression in three out of four MTC patient tissues that had Nav1.7 expression.
